# The Effect of Phantom Stimulation and Pseudomonophasic Pulse Shapes on Pitch Perception by Cochlear Implant Listeners

**DOI:** 10.1007/s10162-020-00768-x

**Published:** 2020-08-17

**Authors:** Wiebke Lamping, John M. Deeks, Jeremy Marozeau, Robert P. Carlyon

**Affiliations:** 1 Hearing Systems Section, Department of Health Technology, Technical University of Denmark, DK-2800 Kgs. Lyngby, Denmark; 2 Medical Research Council Cognition and Brain Sciences Unit, University of Cambridge, 15 Chaucer Road, Cambridge, CB2 7EF UK; 3 Department of Clinical Neurosciences, University of Cambridge, Cambridge, CB2 0SZ UK

**Keywords:** cochlear implants, pitch perception, phantom stimulation, asymmetric pulses

## Abstract

It has been suggested that a specialized high-temporal-acuity brainstem pathway can be activated by stimulating more apically in the cochlea than is achieved by cochlear implants (CIs) when programmed with contemporary clinical settings. We performed multiple experiments to test the effect on pitch perception of phantom stimulation and asymmetric current pulses, both supposedly stimulating beyond the most apical electrode of a CI. The two stimulus types were generated using a bipolar electrode pair, composed of the most apical electrode of the array and a neighboring, more basal electrode. Experiment 1 used a pitch-ranking procedure where neural excitation was shifted apically or basally using so-called phantom stimulation. No benefit of apical phantom stimulation was found on the highest rate up to which pitch ranks increased (upper limit), nor on the slopes of the pitch-ranking function above 300 pulses per second (pps). Experiment 2 used the same procedure to study the effects of apical pseudomonophasic pulses, where the locus of excitation was manipulated by changing stimulus polarity. A benefit of apical stimulation was obtained for the slopes above 300 pps. Experiment 3 used an adaptive rate discrimination procedure and found a small but significant benefit of both types of apical stimulation. Overall, the results show some benefit for apical stimulation on temporal pitch processing at high pulse rates but reveal that the effect is smaller and more variable across listeners than suggested by previous research. The results also provide some indication that the benefit of apical stimulation may decline over time since implantation.

## **INTRODUCTION**

Cochlear implants (CIs) can convey pitch along two orthogonal perceptual dimensions, related to the place of excitation in the cochlea and the temporal pattern of stimulation. However, the perception of pitch by CI users remains limited. The range of place pitches is restricted by shallow insertion depths, while the number of discriminable place pitches is restricted by the number of implantable electrodes and the broad spread of neural excitation along the cochlea. The limitations regarding the perception of temporal pitch are revealed by the finding that the discrimination of changes in the rate of pulses presented to a single electrode by CI listeners is worse than the ability of normal-hearing listeners to detect changes in the fundamental frequencies of bandpass-filtered harmonic complexes (Moore and Carlyon 2005; Stahl et al. 2016; Carlyon et al. 2018). This is true even when those harmonics are unresolved by the peripheral auditory system, such that there are no consistent place-of-excitation cues to pitch. Further, temporal pitch deteriorates for most CI users above a subject- and electrode-dependent “upper limit” that ranges between 200 and 900 pulses per second (pps) and is on average around 300 pps (e.g., Zeng 2002). This upper limit is substantially lower than normal-hearing listeners’ upper limit for complex tones consisting of only unresolved harmonics, which ranges from 600 to 800 pps (Carlyon and Deeks 2002; Macherey and Carlyon 2014).

A study by Middlebrooks and Snyder (2010) suggests that temporal pitch perception by CI listeners might be improved by stimulating neurons that innervate the very apex of the cochlea. When using apical intraneural or ball electrode stimulation, where the electrode was inserted deeply into the apex of the cochlea, feline inferior colliculus (IC) neurons had lower characteristic frequencies, shorter first-spike latencies, lower scattering of first-spike latencies, shorter group delays, and higher limiting rates for phase-locking relative to responses from more basal sites. IC neurons did not show these responses when activating the most apical electrode of an intrascalar electrode array, similar to those normally implanted in human recipients.

It is possible that this high-temporal-acuity brainstem pathway could be triggered in human CI listeners by stimulating more apically than is done with the standard clinical methods of CI stimulation. However, despite multiple studies comparing performance on various tasks across different sites of the electrode array (e.g., Baumann and Nobbe 2004; Kong et al. 2009), we are aware of only two studies investigating whether temporal pitch can be improved by stimulating the very apical cochlear regions. Stahl et al. (2016) reported a significant improvement in rate discrimination performance with low-rate pulse trains (20–104 pps) when stimulating one of the most apical electrodes of the long (31 mm) MED-El standard electrode array, compared to a more basal reference electrode. Macherey et al. (2011) used a different approach, which exploited the fact that CI users are more sensitive to anodic than to cathodic stimulation (Macherey et al. 2006, 2008). They presented trains of pseudomonophasic pulses in bipolar mode to electrodes 1 and 3 of the Advanced Bionics device, such that the more apical electrode (number 1) was activated with a short-high phase in anodic polarity followed by a long-low phase in cathodic polarity. The interaction with the opposite-polarity stimulus presented to electrode 3 was hypothesized to shift the electric field apically relative to electrode 1, potentially allowing the short-high anodic phase to stimulate neurons located more apically than that electrode, while the long-low cathodic phase was assumed to be less effective. When using this type of stimulation, a significant increase in the upper limit of temporal pitch was found (to approximately 700 pps) compared to a polarity-inverted version of that stimulus presented to the same electrode pair or to another electrode pair at a more basal location.

Another method that could selectively excite the cochlear apex is so-called phantom stimulation (Wilson et al. 1992), which, like apical pseudomonophasic-anodic pulse trains, has been shown to elicit a place pitch lower than the most apical electrode in monopolar mode (Saoji and Litvak 2010; Macherey and Carlyon 2012; Saoji et al. 2013). For apical phantom stimulation, current is injected through one electrode of a bipolar pair (primary contact), often the most apical electrode, returning only a proportion through the other electrode (compensating contact), with the remainder returned via an extra-cochlear electrode. Implementing more apical stimulation into contemporary CI processing could potentially extend temporal pitch and transmit more information about the acoustic environment to the CI recipient. This might in turn enhance listeners’ performance, particularly in more complex acoustic scenarios. However, no studies to date have investigated how temporal pitch perception is affected by selectively exciting the apex using phantom stimulation.

In this study, we tested whether phantom stimulation and/or the use of asymmetric pulses could improve pitch perception. The first experiment of this study investigated whether apical phantom stimulation could increase the upper limit of temporal pitch. In experiment 2, we replicated an experiment by Macherey et al. (2011) that examined the effect apical pseudomonophasic-anodic first pulse shapes on the upper limit. Finally, experiment 3 tested both types of apical stimulation on rate discrimination performance, determining thresholds for a high- and a low-rate standard to assess temporal acuity. The results show a higher degree of variability than expected from previous studies. Possible reasons for the variation in the effectiveness of the manipulation across listeners and differences between present and previous results will be discussed.

## **EXPERIMENT 1: PHANTOM STIMULATION**

Experiment 1 investigates the effect of phantom electrode stimulation on the upper limit of temporal pitch. Conditions where the primary contact is the apical or basal member of the bipolar pair will be referred to as apical phantom and basal phantom stimulation, respectively. To test whether apical phantom stimulation can increase the upper limit of temporal pitch, we first performed an experiment to identify the phantom stimulus parameters resulting in the lowest possible place pitch, and hence, presumably, the most apical stimulation. This experiment also identifies those parameters that, when applied to the same pair of electrodes, yield the highest possible place pitch and thus more basal stimulation.

### Place Pitch

#### Overview

**Fig. 1:**
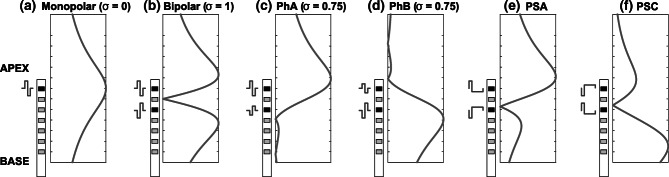
Illustration of the voltage distribution caused by various stimulation techniques, only the positive voltage deflections are shown. Black and gray contacts depict active and inactive electrodes, respectively. Distribution estimates are demonstrated for **a** monopolar, **b** bipolar, **c** apical phantom with *σ* = 0.75, **d** basal phantom with *σ* = 0.75, **e** bipolar pseudomonophasic anodic-first, and **f** bipolar pseudomonophasic cathodic-first stimulation

Figure 1 shows the expected voltage distribution (cf. Rattay 1989) for phantom stimulation and other types of stimulation.

The response to one electrode being active in monopolar (MP) stimulation mode is shown in Fig. 1a. All remaining stimulus configurations (Fig. 1b–f) are in bipolar (BP) stimulation mode, where two intra-cochlear electrodes are activated simultaneously with one inactive electrode between them (BP + 1). For full bipolar stimulation mode (Fig. 1b), the phases of a pulse applied to one electrode are first anodic and then cathodic, while the other electrode is activated with the same current amplitude but in opposite phase. Thus, the excitation caused by both electrodes is assumed to be similar. For partial bipolar or “phantom” stimulation, the current ratio *σ* between the primary and the compensating electrode is altered to shift the locus of excitation further towards, and beyond, the primary electrode (e.g., Townshend et al. 1987). An example of phantom stimulation with the primary electrode defined as the more apical of the bipolar pair (“PhA”) and *σ* = 0.75, pushing the locus of excitation towards the apex, is shown in Fig. 1c. A stimulus with the more basal electrode as the primary electrode (“PhB”) and *σ* = 0.75, pushing the locus of excitation towards more basal sites, is shown in Fig. 1d.

#### Subjects

Eleven CI listeners took part, all of them users of the CII/HiRes 90k CI device manufactured by Advanced Bionics. Their demographic data can be found in Table 1. Data were collected in both Cambridge (UK) and Lyngby (DK). The research was approved by the National Research Ethics Committee for the East of England (Ref. No. 00/327) and by the Science-Ethics Committee for the Capital Region of Denmark (reference H-16036391). All listeners provided written informed consent prior to participation in the experiment. Experiments were carried out using custom Matlab (The MathWorks, Inc., Natick, MA, USA) experimental interfaces which had been modified to interact with the Bionic Ear Data Collection System (BEDCS, Advanced Bionics, Litvak 2003). Impedances for each subject were measured prior to testing, as well as at the end of each experimental session, which is a standard safety check. One benefit of this check is that it allowed us to ensure that the voltage requirements were always kept below the compliance limit for electrical stimulation. Subjects were paid and travel expenses were reimbursed.

**Table 1:** Details of the subjects who took part in the experiments. Subjects were either implanted in the UK and tested in Cambridge (UK) or implanted in Denmark and tested in Lyngby (DK). Information regarding the duration of deafness was not available (N.A.) for all listeners

Listener	Sex	Age	Onset of hearing loss	Implant type	Country	Years of implant use	Duration of deafness
AB01	M	73	Post-lingual, progressive	HR90k Advantage/HiFocus MS	UK	9	N.A.
AB02	F	50	Post-lingual, progressive	HR90k/HiFocus 1J	UK	10	16
AB03	M	71	Post-lingual, progressive	HR90k/HiFocus 1J	UK	9	N.A.
AB05	M	75	Post-lingual, progressive	HR90k/HiFocus 1J	UK	7	30
AB06	F	68	Peri-lingual, progressive	HR90k/HiFocus 1J	UK	3	25
AB09	M	71	Post-lingual, progressive	HR90K Advantage/HiFocus MS	UK	4	N.A.
AB13	M	78	Post-lingual, progressive	HR90k/HiFocus 1J	UK	10	10
AB24	F	47	Post-lingual, sudden	HR90k Advantage/HiFocus MS	UK	1	1
AB27	M	78	Post-lingual, progressive	HiRes Ultra/HiFocus ms	UK	0.5	30
S1-DK	F	27	Post-lingual, progressive	HR90k/HiFocus 1J	DK	4	4
S2-DK	F	60	Post-lingual, progressive	HR90k/ HiFocus 1J	DK	8	N.A.

#### Methods

This part of the experiment estimated the value of *σ* that yielded the lowest place pitch for PhA stimulation and the value of *σ* that yielded the highest place pitch for PhB stimulation as a control condition, using a similar procedure to Macherey and Carlyon (2012). Stimuli were 500-ms charge-balanced biphasic pulse trains delivered at a rate of 20 pps on electrodes 1 (E1) and 3 (E3), with a phase duration of 97 μs and no inter-phase gap. These low-rate pulse trains were used to avoid any influence of temporal pitch on listeners’ place-pitch judgments (Carlyon et al. 2010). For PhA, five stimuli were constructed having different current ratios *σ*, ranging from *σ* = 0 (monopolar mode, with case electrode serving as ground) to *σ* = 1 (bipolar mode) in steps of 0.25. For PhB, the *σ* values ranged from 0.25 to 1 in steps of 0.25.

Prior to the place-pitch measurements, the stimuli were loudness balanced. First, the most comfortable level (MCL) was determined for each stimulus; the current level was gradually increased starting from zero, while listeners indicated the loudness level using a chart that was marked on a scale from 0 (“off”) to 10 (“too loud”). Once loudness level 7 (“loud but comfortable”) was reached, the stimulus level was reduced until loudness level 6 (MCL) was confirmed. Second, the stimuli were loudness balanced. The balancing procedure was based on that proposed by McKay and McDermott (1998) and similar to the one used by Carlyon et al. (2018), presenting one stimulus fixed in level (standard) followed by a second stimulus to be adjusted (signal). After each presentation of the pair, the subject adjusted the loudness of the stimuli by either increasing or decreasing the level of the signal until they were perceived as equally loud. The comparisons were performed twice and the average of the obtained signal current levels was taken as the matched level. Next, standard and signal were swapped, and the previously matched level was presented as the new standard level. This procedure was again repeated twice, and the log-transformed average difference was calculated to be the loudness balanced level for the signal. This way, each (partial) bipolar stimulus was balanced to the monopolar reference (*σ* = 0) on E1 set to its MCL.

The loudness-balanced stimuli were then ranked in pitch using the midpoint comparison procedure (MPC, Long et al. 2005), which is based on a series of two-alternative forced choice (2AFC) trials. During each trial, two stimuli were presented with a 500-ms gap and the subject had to indicate which one had the higher pitch. The selection of stimuli to be used in each consecutive trial was based on the results of the previous trial so that the total number of comparisons was minimized. No feedback was given and the whole procedure was repeated 20 times. Two separate blocks of the MPCs were performed to identify the ratio eliciting the lowest pitch in the PhA condition and the ratio eliciting the highest pitch in the PhB condition. As noted above, the ratios used for PhB ranged between 0.25 and 1 but the MPC also included the PhA stimulus that elicited the lowest pitch previously obtained for the respective listener. This set of stimuli was chosen to confirm that, for each participant, the highest pitch obtained with PhB was higher than the lowest pitch obtained with PhA.

It is possible that the whole rank order of one run could be affected by an erroneous trial. For this reason, a later analysis removed entire runs where any individual rank exceeded the mean rank across runs for that stimulus by ± 2.6 times the standard deviation (99 % confidence interval). For PhA, maximally 3 out of the 20 runs were removed for a given individual, while for PhB, maximally 4 out of 20 runs were removed.

Statistical analysis was performed by fitting a linear mixed-effects model to the mean pitch ranks with current ratio as fixed effect, treated as a categorical variable, and test subject as random effect. The model was implemented in R (R Core Team 2015) using the lme4 package (Bates et al. 2015). Model selection was performed with the lmerTest package (Kuznetsova et al. 2017), following the backward selection approach which is based on stepwise deletion of model terms with high *p* values (Kuznetsova et al. 2015). *P* values for the fixed-effects term were calculated from *F* tests (Satterthwaite’s approximation of dominator degrees of freedom) while *p* values for the random effects were calculated based on likelihood ratio tests (Kuznetsova et al. 2015). Post hoc analysis was performed using the multcomp package (Hothorn et al. 2008), and the *p* values were corrected for multiple comparisons using Tukey’s range test unless stated otherwise.

#### Results and Discussion

**Fig. 2:**
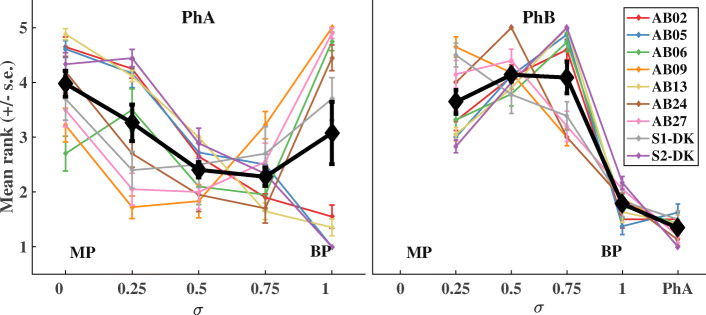
Place-pitch ranking results for PhA (left panel) and PhB (right panel) stimuli. The mean pitch rank and standard error are shown in black as a function of the ratio of current returning to the compensating electrode, from monopolar stimulation (*σ* = 0) to bipolar stimulation (*σ* = 1). Colored lines depict results from individual listeners

The left and right panels of Fig. 2 show individual (colored lines) and mean pitch ranks (black diamonds) as a function of *σ*, for the PhA and PhB stimuli respectively. Error bars show the standard errors of pitch ranks across listeners. Table 2 shows the ratios eliciting the lowest and highest pitch per condition for each individual listener.

**Table 2:** Current ratio eliciting the highest and lowest pitch for PhA (top) and PhB (bottom) for each individual listener

Subject ID	AB02	AB05	AB06	AB13	AB09	AB24	AB27	S1-DK	S2-DK	Mean
PhA lowest *σ*	1	1	0.75	1	0.25	0.75	0.25	0.25	1	0.69
PhB highest *σ*	0.75	0.75	0.75	0.75	0.25	0.5	0.25	0.25	0.75	0.56

**Fig. 3:**
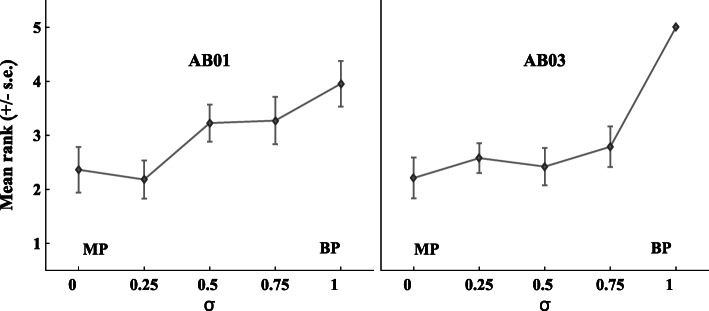
Individual place-pitch ranking results for listeners AB01 (left panel) and AB03 (right panel). Mean pitch rank and standard error are shown as a function of the current ratio *σ*. For these two listeners, pitch ranking was only performed for the PhA stimulus condition

For two of the listeners (AB01 and AB03), the place pitch could not be successfully lowered relative to the most apical electrode in MP mode. Their results can be seen in Fig. 3. Similar observations have been made before with phantom electrode stimulation (e.g., Macherey and Carlyon 2012), but reasons for a failure to lower pitch relative to the most apical electrode in monopolar mode remain unclear. Arguably, they may be caused by a place- pitch reversal where E1 is perceived as higher in pitch than E3, but additional experimental work would be needed to confirm this. Listeners AB01 and AB03 were excluded from the statistical analysis, as well as from further testing.

For PhA, the average results are in agreement with previous findings (Saoji and Litvak 2010; Macherey and Carlyon 2012) while the individual results show the expected variability as the ratio eliciting the lowest pitch ranged between *σ* = 0.25 and *σ* = 1. The statistical analysis indicated that pitch ranks were significantly affected by *σ* for PhA (*F*(4,40) = 3.99, *p* < 0.01) and for PhB (*F*(4,40) = 51.88, *p* < 0.01). Finally, post hoc comparisons showed that a significant difference (*p* < 0.005) could be achieved between the PhA condition eliciting the lowest pitch and the PhB condition eliciting the highest pitch (Fig. 2, right).

Differences in neural survival, and/or the distance between electrodes and the modiolus, determine the differences in sensitivity to the current on the compensating electrode across listeners, which in turn may affect the *σ* leading to the lowest or highest pitch. Further, neural survival has been shown to be better at apical than basal sites (e.g., Bierer 2007) and may explain why the *σ* leading to the lowest pitch for PhA reached 1 in some cases. A *σ* of 1 will cause the maximum possible apical shift of E1’s activation pattern, and, where neural survival is supposedly weaker on E3, the effect on pitch will not be counteracted by the basalward shift in E3’s activation pattern. This explanation is also consistent with the fact that the *σ* leading to the highest pitch for PhB stimulation was never as high as 1 (see Fig. 1d). This is because PhB stimulation with *σ* = 1 would have presented substantial excitation near the compensating electrode (E1), where neural survival is likely to be high.

### Temporal Pitch

This part of the experiment measured whether the upper limit of temporal pitch can be increased by apical phantom stimulation. To do so the upper limit was measured for an MP stimulus on E1 and for PhA and PhB stimulation, using the listener-specific values of *σ* that produced the lowest and highest place pitch as described above.

#### Methods

An MPC procedure, similar to the one used by Macherey et al. (2011), was implemented. It involved pitch-ranking eight different pulse rates, which were logarithmically spaced from 142 to 1159 pps with a 35 % difference between consecutive rates. Apart from the different pulse rates and current levels, the stimulus parameters (stimulus duration, phase duration, inter-phase gap, and electrode position) for MP, PhA, and PhB stimuli were the same as for the place-pitch measurements. For the PhB condition, no benefit would be expected but it was included in the experiment to serve as a control condition as other cues inherent to phantom stimulation may help listeners to discriminate systematically between the stimuli. Eight subjects (AB02, AB05, AB06, AB13, AB24, AB27, S1-DK, S2-DK) participated.

All stimuli were loudness-scaled to their respective MCL before loudness balancing them. Balancing was carried out in a manner similar to the place-pitch experiment: First, the MP stimulus at 142 pps was set to its MCL and used as the reference stimulus, and the PhA and PhB stimuli at 142 pps were each loudness balanced to it. Second, for each stimulus condition, higher rates were balanced within their respective condition for four out of the 8 rates between 142 and 1159 pps, i.e., within each condition, the 259 pps was balanced to the 142 pps, the 471 to the 259 pps and the 1159 pps to the 471 pps stimulus. Loudness levels for intermediate rates were logarithmically interpolated.

**Fig. 4:**
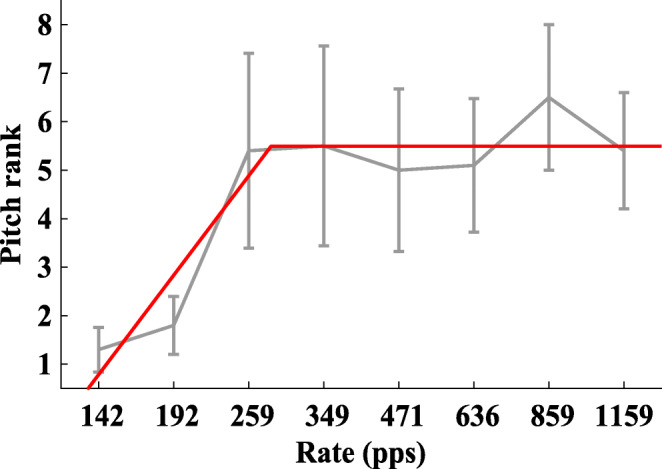
Example of an MPC pitch-ranking function, including the broken-stick fit for that respective ranking result

The upper limit of temporal pitch was determined in three separate MPC blocks for the three different stimulus conditions. In each block, ten repetitions were conducted to obtain the mean rank and standard error. A broken-stick function was fitted to the MPC, using the Matlab Curve Fitting Toolbox. The pulse rate at which a breakpoint was estimated was taken as the upper limit. The fitting parameters for the broken stick were identical to those used by Carlyon et al. (2018). An example MPC including the broken stick fit can be seen in Fig. 4.

#### Results and Discussion

**Fig. 5:**
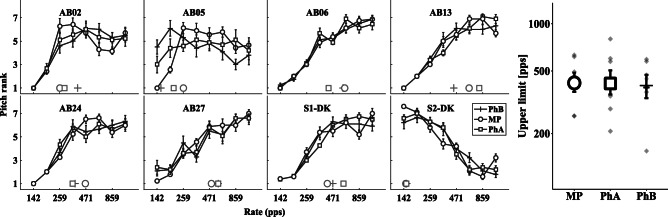
Left: individual pitch ranking results for conditions PhA, PhB, and MP showing the mean rank and standard error as a function of pulse rate. Right: mean and standard error of the upper limit estimate for each condition averaged across participants

The left part of Fig. 5 shows the individual pitch ranking results as a function of pulse rate for MP, PhA, and PhB while the upper limits for each condition are summarized on the right-hand side of the figure. Error bars depict the standard error. The open circles represent the pitch-rank functions for the MP condition, white squares for PhA, and black crosses for PhB. For each participant, gray circles, squares and crosses at the bottom of each graph indicate the respective upper limit estimate.

Individual results generally show a subject-dependent increase in pitch rank with increasing pulse rate, as expected from previous studies (e.g. Kong et al. 2009). However, subject AB05 showed a flat MPC for both phantom conditions and reported difficulties in perceiving differences in pitch for these stimuli. This subject also had relatively high *σ* values needed to achieve the lowest and highest pitch (PhA = 1, PhB = 0.75) and large differences in MCL between the MP and PhA condition (4.38 dB) and the MP and PhB condition (3.77 dB), as shown in Table 3. For S2-DK, a decrease in pitch with an increase in rate was observed. As this subject did not confuse “low” and “high” in the place pitch measurements, i.e. showed a shift in the expected direction, their results may reflect a temporal pitch reversal. These reversals have been reported previously (Kong and Carlyon 2010; Cosentino et al. 2016), although their basis is unknown. Due to her markedly atypical pitch ranking results, S2-DK was excluded from further testing and from the statistical analysis.

**Table 3:** Upper limits, slopes after 300 pps, and MCLs (in dB re 1 mA) for each individual participant comprising monopolar (MP) stimulation on E1 and phantom stimulation on E1 and E3 shifting the locus of excitation apically (PhA) or basally (PhB)

Analysis	Condition	AB02	AB05	AB06	AB13	AB24	AB27	S1-DK
Upper limits	MP	259	259	615	631	463	493	412
	PhA	287	207	426	798	346	574	599
	PhB	392	154	592	445	366	575	470
Slopes	MP	− 0.27	0.27	− 0.27	0.04	0.27	0.54	0.5
	PhA	− 0.10	0.68	− 0.10	0.17	0.39	0.30	0.42
	PhB	− 0.02	0.14	− 0.27	0.08	0.06	0.48	0.51
MCL	MP	− 8.41	− 9.91	− 6.31	− 7.74	− 6.65	− 7.03	− 7.59
	PhA	− 2.01	− 5.53	− 2.56	− 1.61	− 3.60	− 4.33	− 6.99
	PhB	− 3.68	− 6.14	− 3.12	− 2.71	− 4.96	− 5.11	− 5.92

A linear mixed-effects model was fitted to the log-transformed upper limit with stimulus condition as fixed effect and test subject as random effect. No significant main effect of condition on the upper limit of pitch was found (*F*(2,12) = 0.17, *p* = 0.84). Macherey et al. (2011) also showed that the slopes above 300 pps (typical upper limit) differed significantly between conditions, with apical stimulation causing significantly steeper increase than the other conditions tested. However, fitting a linear mixed-effects model to the slopes after 300 pps for the MP, PhA, and PhB condition revealed no significant main effect of condition either (*F*(2,12) = 1.18, *p* = 0.34). The estimated upper limit, slopes, and MCLs for each participant can be found in Table 3.

This result was surprising as Macherey et al. (2011) showed a significant increase in the upper limit using asymmetric pulses to produce selective apical stimulation, and because phantom stimulation has been shown to produce similar shifts in the place of excitation to that method (Macherey and Carlyon 2012). Possible discrepancies between the present and previous experiments may be related to inter-subject differences. Alternatively, the different types of stimulation across studies, i.e., phantom stimulation and pseudomonophasic pulse shapes, could have had an influence on the results. It is possible that PhA stimulation with an average ratio of *σ* = 0.69 would generate a side lobe large enough to cause neurons close to E3 to be recruited, potentially leading to less-reliable pitch coding arising from more-basal sites. To be able to compare the effect of both types of stimulation on the upper limit in the same set of listeners, the experiment by Macherey et al. (2011) using pseudomonophasic pulse shapes was replicated and is shown and discussed below.

## **EXPERIMENT 2: PSEUDOMONOPHASIC STIMULATION**

To our surprise, experiment 1 did not reveal an effect of apical phantom stimulation on the upper limit of temporal pitch. Given that Macherey et al. (2011) reported a significant increase in the upper limit when shifting the locus of excitation towards more apical sites using pseudomonophasic pulses, their experiment was replicated here using the same set of subjects as for experiment 1. This was done so as to test whether the lack of a benefit may stem from differences between types of stimulation and to confirm (or otherwise) the previous findings from Macherey et al. (2011). Again, the procedure was carried out in two steps: First, place pitch comparisons were conducted to confirm a place pitch shift towards the apex. Second, the upper limit was obtained using the same pitch ranking procedure as previously described.

The effect of presenting pseudomonophasic stimuli in bipolar mode on the locus of excitation is illustrated in Fig. 1e, f. Note that the graph illustrates the positive voltage deflection because, for human listeners, anodic currents are more effective than cathodic currents in eliciting comfortable loudness and in exciting the auditory nerve (Macherey et al. 2006, 2008; Undurraga et al. 2010, 2013). Another reason why a shift in neural excitation can be achieved with these stimuli is that, for equal charge, shorter phases are more effective than longer phases (Shannon 1985; Pfingst and Morris 1993; McKay and McDermott 1998). Accordingly, pseudomonophasic anodic-first (PSA) stimuli (Fig. 1e), where the short-high anodic phase is presented to the more apical electrode, have been shown to produce lower place pitches than pseudomonophasic cathodic-first (PSC) stimuli (Fig. 1f), where the short-high anodic phase is presented to the more basal electrode (Macherey et al. 2011; Macherey and Carlyon 2012).

### Place Pitch

#### Rationale and Methods

This part of the experiment compares the place pitches of 500-ms trains of PSA and PSC pulses to each other and to a symmetric pulse train presented to E1 in monopolar (MP) mode. The duration of the first phase of each pulse was 97 μs. For pseudomonophasic pulse trains, the duration of the second phase was increased by a factor of four; to keep the stimulus charge balanced, the amplitude of that phase had to be reduced by the same factor. To prevent the subjects from overlearning the stimuli, the place-pitch comparisons were conducted using two different pulse rates, 20 and 33 pps, which were presented in a randomly intermixed fashion. The place-pitch comparisons between PSA, PSC, and MP were assessed in a 2AFC task where two randomly chosen stimuli were presented at the same rate and the listener was asked which stimulus had the higher pitch. Two blocks of 60 trials were performed leading to a total of 20 repetitions per comparison. No feedback was provided. Six CI listeners (AB02, AB05, AB06, AB13, AB24, AB27) participated. Prior to testing, the stimuli were loudness-scaled and balanced as described above, with PSA and PSC stimuli being balanced to the monopolar reference stimulus at their respective rate.

#### Results and Discussion

**Fig. 6:**
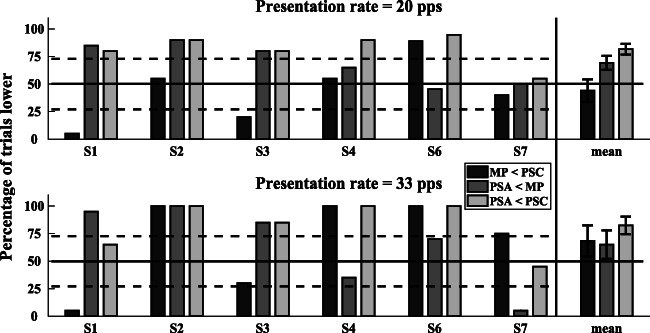
Place pitch comparisons for MP, PSA, and PSC. Legend shows percentage of times condition B was judged as higher in pitch than A (A < B). Individual results are displayed, as well as mean and standard error on the utmost right of the graph. The error bars show the 95 % confidence intervals, the dashed lines at 28 and 72 % show the 95 % confidence interval, and the solid line indicates chance level. Top and bottom panels show results for the two different stimulation rates of 20 and 33 pps, respectively

The percentage of trials where one stimulus was judged as lower in pitch than the one it was compared to can be seen in Fig. 6. For instance, “PSA < PSC” with 75 % would indicate that PSA was judged lower in pitch than PSC on 75 % of the trials. Results are shown for individual listeners on the left and for the group on the right. The solid line indicates chance level. The dashed lines at 28 and 72 % show the 95 % confidence interval based on a binomial distribution with *p* = 0.5 (two-sided). The error bars in the right-hand part of the graph depict the across-subject standard errors. Upper and lower panels show results for the two different rates of 20 and 33 pps, respectively.

For each comparison separately, a linear mixed model was fitted to the percent correct scores with participant as random effect and pulse rate as fixed effect. No significant difference between rates was found for comparisons of either “MP < PSC” (*F*(1,4.9) = 0.84, *p* = 0.40) or “PSA < MP” (*F*(1,5) = 0.005, *p* = 0.9) or “PSA < PSC” (*F*(1,5) = 0.006, *p* = 0.9). For this reason, the 95 % confidence intervals were calculated for each comparison combining both rates. This analysis, which was conducted so as to compare to Macherey et al. (2011), revealed that the confidence intervals for “PSA < PSC” [70.47, 93.69] fell outside the 50 % level (see Fig. 6), and therefore showed a significant difference between the pitches of the PSA and PSC stimuli.

Nevertheless, the comparisons between the MP condition and each of the two pseudomonophasic conditions (PSA, PSC) did not differ significantly from chance. This may have been due to small differences in neural survival preventing the pseudomonophasic stimuli from selectively recruiting neurons near the active electrode. It is worth noting that, although Macherey et al. (2011) observed larger average differences than reported here, they too found that not all listeners showed a consistent pitch shift for pseudomonophasic stimuli in the expected direction relative to the MP reference. However, PSA led to a significantly lower pitch and thus presumably more apical stimulation relative to PSC and so differences in temporal pitch could still be expected between the two conditions.

### Temporal Pitch

#### Rationale and Methods

The effect of pseudomonophasic pulses on the upper limit of temporal pitch was investigated using the same experimental procedure as outlined in experiment 1 but with pseudomonophasic pulse shapes (on E1 and E3) as described in the place-pitch section of this experiment. Seven subjects (AB02, AB05, AB06, AB13, AB24, AB27, S1-DK) participated.

As a possible predictor for the variability observed in the upper limit data, the state of the electrode neuron interface was assessed for each listener by obtaining detection thresholds on E1 and E3 through an adaptive procedure. Stimuli were 300-ms symmetric, biphasic pulse trains in MP mode with a pulse rate of 80 pps and a phase duration of 43 μs. The procedure and parameters were selected as low-rate detection thresholds have been proposed in numerous studies to predict neural health (Zhou and Pfingst 2016; Zhou 2017; Zhou and Dong 2017). The stimuli were first presented at a “soft” level, which was decreased after every correct trial and increased by the same amount after every incorrect trial. The change from decreasing to increasing current or vice versa defined a reversal. The step size was 0.5 dB before the first reversal and 0.2 dB thereafter. The procedure stopped after eight reversals, and the final value for the track was determined from the mean (in dB) of the last six reversals. Four runs were conducted and the last three were averaged and taken as threshold.

#### Results and Discussion

**Fig. 7:**
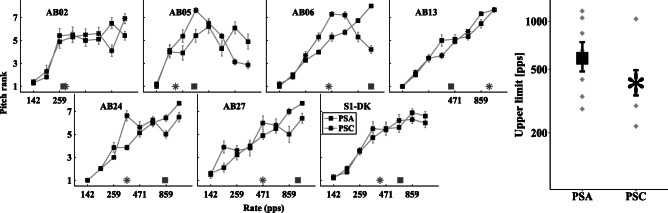
Left: individual pitch ranking results for conditions PSA and PSC showing the mean rank and standard error as a function of pulse rate. Right: mean and standard error of the upper limit estimate for each condition averaged across participants

Figure 7 shows the pitch-rank functions for each subject on the left, with upper limits for each individual and for the mean on the right. Black squares represent the PSA condition, black asterisks the PSC condition, and gray squares and asterisks at the bottom of the graph the respective upper limit estimate for each participant.

As in exp. 1, the individual data showed an increase in pulse rate leading to an increase in perceived pitch up to some subject-specific rate. A considerable across-subject variability can be observed, particularly with respect to the effect of stimulus condition as some subjects show no benefit of more apical stimulation using PSA pulses (AB02, AB13), some show a small benefit (AB05, S1-DK), and some show a large benefit (AB06, AB24, AB27).

On average, PSA stimulation led to a higher upper limit (600 pps) compared to PSC (412 pps) stimulation. However, and unlike the results of Macherey et al. (2011), this difference was not significant when using a linear mixed-effects model for analysis with the log-transformed upper limit as dependent variable, participant as random effect, and condition as fixed-effect term (*F*(1,6) = 2.34, *p* = 0.17).

As noted previously, Macherey et al. (2011) also estimated the upper limit of pitch using the slope of the MPC function above 300 pps. A mixed model fitted to the slopes with condition as fixed effect and test subject as random effect did reveal a significant main effect (*F*(1,11.31) = 34.99, *p* < 0.0005). To test whether the way pitch ranks vary with rate differs across conditions, a mixed model was fitted to the MPC ranks with stimulus condition and pulse rate as fixed effects and subject as a random effect. This revealed a significant interaction effect between condition and rate (*F*(7,96) = 2.75, *p* < 0.01209), and Bonferroni-corrected post hoc comparisons showed a significant difference between PSA and PSC at a rate of 859 pps (*p* < 0.0388). Table 4 shows the slopes as well as the estimated upper limit for each individual listener across conditions.

**Table 4:** Upper limits, slopes after 300 pps, and MCLs (in dB re 1 mA) for each individual participant for pseudomonophasic pulses on E1 and E3, shifting the locus of excitation apically (PSA) or basally (PSC)

Analysis	Condition	AB02	AB05	AB06	AB13	AB24	AB27	S1-DK
Upper limits	PSA	283	338	1158	433	840	587	656
	PSC	296	220	440	1034	350	471	410
Slopes	PSA	0.11	− 0.07	0.90	0.64	0.68	0.85	0.45
	PSC	0.15	− 0.95	− 0.38	0.93	0.00	0.28	0.10
MCL	PSA	− 1.97	− 4.81	− 1.27	− 1.72	− 0.84	− 3.63	−5.87
	PSC	− 1.99	− 3.28	− 1.37	− 0.32	− 0.52	− 2.46	−6.88

Unlike results of Macherey et al. (2011), there was no significant correlation between the slopes of the MPC functions and the MCLs (*r* = 0.08, *p* = 0.85). Detection thresholds for low-rate pulse trains were obtained as they have previously been proposed as a more thorough predictor of the electrode-neuron interface than comfort levels (Zhou and Pfingst 2016; Zhou 2017; Zhou and Dong 2017). Still, the threshold difference between E1 and E3 (in decibels re to 1 mA) was not significantly correlated with the difference between PSA and PSC for the log-transformed upper limit (*r* = 0.31, *p* = 0.49) or slopes (*r* = 0.08, *p* = 0.85). Thus, the across-subject variability obtained in experiment 2 cannot be explained by the estimates of neural survival used here.

## **EXPERIMENT 3: EFFECT OF PHANTOM STIMULATION AND PSEUDOMONOPHASIC PULSES ON RATE DISCRIMINATION RATIOS**

### Rationale and Method

Experiment 2 showed that the effect of apical stimulation seemed to depend on the outcome measure, as PSA pulse trains produced a significantly steeper growth of the pitch-ranking function above 300 pps, while showing no effect on the upper limit when fitted with a broken stick. Despite the high test-retest reliability of the broken-stick estimate (Carlyon et al. 2018), one reason for this may be that the MPC functions shown in Fig. 7 sometimes deviated from the canonical shape. For example, two subjects, AB05 and AB06, showed pitch reversals in the PSC condition, and the only subject (AB13) whose upper limit was lower for PSA than for PSC showed an MPC function that reached a plateau at 349 pps and then rose again above 636 pps. Experiment 3 therefore measured temporal processing using a different method, as proposed by Cosentino et al. (2016), for both phantom stimulation and pseudomonophasic pulses. This method involves measuring the smallest detectable difference in pulse rate (just noticeable difference, JND), expressed as a ratio, relative to either a low-rate or a high-rate standard. For the lower-rate standard, this “rate discrimination ratio (RDR)” was obtained with the signal higher in rate than the standard. This lower rate, which was 80 pps, was included in the procedure because the high-temporal-acuity pathway found by Middlebrooks and Snyder (2010) might also be reflected by generally better discrimination performance, even for lower rates. For the higher-rate standard, the signal rate was lower than the 600-pps standard, and can be seen as an alternative estimate of the upper limit of pitch. The rationale is that the 600-pps standard is higher than the upper limit for most subjects and that the RDR reflects the highest rate above which the rate-pitch function asymptotes. An advantage of this method is that it returns an objective and unambiguous measure of the RDR and allows one to study sensitivity to rate changes at a much finer scale than is usually obtained from a pitch ranking procedure. A potential disadvantage is that it is not suitable for instances of pitch reversals.

RDRs were measured for six subjects (AB06, AB13, AB02, AB05, AB24, AB27) using the stimulus conditions MP, PhA, PhB, PSA, and PSC, as defined in experiments 1 and 2. RDRs for the low-rate standard were calculated by dividing the measured rate detection threshold (RDT) by the standard while RDRs for the high-rate standard were calculated by dividing the standard by the RDT. This was done so that, for both standards, low RDRs corresponded to better performance (see Cosentino et al. 2016).

The stimuli were loudness balanced as follows. First, 500-ms 80-pps pulse trains were loudness scaled in the PSA, PSC, PhA, PhB, and MP conditions. Second, these stimuli were then balanced to the 142-pps trains at comfort level that were used in experiment 1 and 2 within their respective condition. The comfort levels for rates between 142 and 1159 pps that had been determined previously were reconfirmed and kept.

The task was a 2AFC with 1 up 3 down–tracking procedure, and the subjects had to indicate which stimulus had the higher pitch by pressing one of the two virtual buttons displayed on a computer screen. Correct-answer feedback was provided after each trial. The choice of which standard to use was made at random from trial to trial. The starting point for both tracks was 150 pps so as to be sufficiently below the upper limit and avoid non-converging tracks. Hence, for the first trial of the lower track subjects compared an 80-pps standard to a 150-pps signal, and the first trial of the upper track involved a comparison between 150 pps and 600 pps. The rate difference between signal and standard rate was adjusted by a factor of 1.5 initially and was then reduced to 1.1 after the first two reversals. The procedure stopped after eight reversals, and the threshold was determined as the geometric mean of the last six reversals. Three runs were obtained for each stimulus condition, and the geometric mean of ratios across runs was taken as the final RDR.

### Results and Discussion

**Fig. 8:**
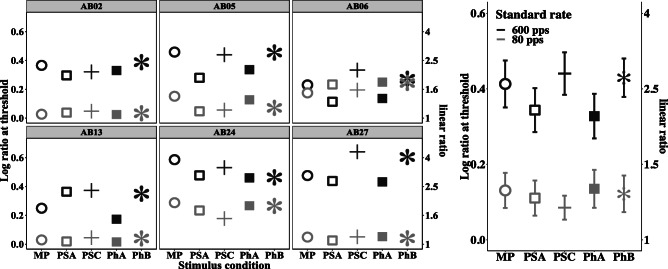
Log (left ordinate) and linear (right ordinate) rate discrimination ratios showing individual (left panel) and average (right panel) performance for all five conditions with standard rates of 80 pps (gray squares) and 600 pps (black circles). Error bars depict the standard error

Figure 8 shows the obtained RDRs across the different stimulus conditions for each individual listener (left) and for the mean results (right). The left and right ordinates show log-transformed and linear RDRs, respectively; all analyses were performed on the log RDRs, but the linear data are also presented for ease of interpretation. As before, white circles show results for MP, white squares for PhA, black crosses for PhB, black squares for PSA, and black asterisks for PSC stimuli. The high-rate standard RDRs are represented in black and the low-rate RDRs in gray. Error bars depict the standard error.

Similar to experiment 1 and 2, the data show a high across-subject variability, particularly for the high-rate RDRs. The average RDRs for the high-rate standard are higher (worse) than for the low-rate standard, in agreement with results from previous studies (McKay et al. 1994; Baumann and Nobbe 2004; Cosentino et al. 2016). Average low- and high-rate RDRs are similar or slightly worse than those reported previously (Baumann and Nobbe 2004; Stahl et al. 2016). Differences in results may occur due to slightly different standard rates and different methods used to obtain thresholds, the amount of training provided, or inter-subject differences.

Fitting a linear mixed-effects model to the RDRs with condition and standard (low or high rate) as fixed effects, including their interaction, and test subject as random effect showed that there was a significant main effect of the standard rate (*F*(1,53) = 94.989, *p* < 0.005).

Post hoc testing with uncorrected *p* values were used for this experiment because the correction for multiple comparisons can arguably be omitted due to conditional testing (Althouse 2016). This analysis revealed no significant differences between any of the conditions for the low-rate standard. For the high-rate standard, the analysis showed significantly smaller RDRs (corresponding to a higher upper limit) when comparing PSA to PSC (*p* = 0.041), as well as PhA to PhB (*p* = 0.037). A test of the overall effect of apical stimulation, using orthogonal contrasts and combining PhA and PSA versus PhB and PSC, revealed a *p* value of 0.5 (*b* = 0.275, *t*(20) = 2.655). Neither PhA nor PSA was significantly different to the MP reference.

When looking at the relationship between upper limit (experiment 2) and RDRs for each condition, no significant correlation could be found. This was slightly surprising because Cosentino et al. (2016) found a strong correlation between the two tasks. However, in both studies, the number of listeners was relatively small. The lack of a correlation between MPC and rate discrimination might be related to differences between tasks comparing large or small separations in pulse rate, the randomly chosen stimuli, the tracking procedure, or whether feedback is provided.

## **DISCUSSION AND CONCLUSION**

### Comparison to Previous Studies

To our knowledge, only two previous studies have investigated the effect of very apical stimulation on temporal processing. One study showed better rate discrimination performance using apical stimulation with low-rate stimuli (Stahl et al. 2016). Our results, however, show no effect of apical stimulation for either phantom stimulation or PSA stimuli on low-rate RDRs. One reason for the different findings may be that previously, discrimination performance was compared between a very apical and a very basal electrode with the standard long MED-EL electrode array (Stahl et al. 2016). It is possible that differences in performance with such low rates may only emerge when very distinct populations of neurons are stimulated.

The other study looked at the effect of asymmetric pulse trains on the upper limit of temporal pitch (Macherey et al. 2011). Our replication of this study did not show a significant effect on the upper limit estimated by a broken stick fit, in contrast to their findings. However, and as mentioned above, an alternative assessment of the upper limit (slopes after 300 pps) revealed a significant effect. It might also be possible that the lack of significance when using the broken stick estimate could be related to the relatively small sample size, which should be considered for future research. The pertinent question, however, is not whether the two studies revealed effect sizes that fell on opposite sides of the criterion used to assess significance. Rather, it is important to know, given all the information now available, whether a significant effect still exists. We therefore combined the results of experiment 2 with those obtained by Macherey and colleagues, i.e., PSA-Apex and PSC-Apex (S1 - S6, Macherey et al. 2011), and performed a statistical analysis with the two fixed effects of stimulus condition and experiment (present and previous) and the random effect of test subject. This showed a significant main effect of stimulus condition on the upper limit (*F*(1,11) = 8.87, *p* < 0.01255). Further, the fixed term “experiment” in the linear mixed model was not found to be significant (*F*(1,11) = 2.36, *p* = 0.15), indicating that, even though the effect of PSA stimulation was not found to be significant in the present experiment and significant in the previous one, there is no significant difference between the two datasets. No significant interaction effect was found either (*F*(1,11) = 0.29, *p* = 0.59). Hence the best evidence at present shows that there is indeed a significant benefit of selective apical stimulation on temporal pitch perception, albeit more variable across subjects than previously thought.

### Across-Subject Variability for PSA Stimulation

The across-subject variability in the effect of PSA stimulation on the upper limit was especially large. Possible reasons will be discussed in the following.

First, the differences in temporal pitch could not be explained by differences in the effective place pitch shift measured in experiment 2: when investigating the relationship between the place pitch difference “PSA < PSC” and the difference of the log-transformed upper limit of PSA and PSC by computing the Spearman’s correlation coefficient, no significant correlation was found for either 20 pps (*r*_s_ = 0.35, *p* = 0.49) or 33 pps (*r*_s_ = −0.27, *p* = 0.6). As mentioned above, Macherey et al. (2011) noted a negative correlation between the MCL in decibels re 1 mA and the log-transformed upper limit, which they interpreted as evidence that the upper limit might depend on more peripheral factors, such as neural survival, rather than on apical stimulation per se. However, no significant correlation between the difference of the log-transformed upper limits of PSA and PSC and the dB difference of the PSA and PSC MCLs was found here (Pearson’s *r* = 0.31, *p* = 0.49).

It is worth noting that two of the listeners (AB02 and AB13) did partake in both studies (referred to as S1 and S2 by Macherey et al. (2011)). Despite the upper limit being substantially higher for PSA than PSC stimuli in the previous study, these listeners showed no benefit for PSA over PSC stimulation in the present study. Between the two experiments, the upper limit using PSA stimuli dropped by approximately 400 pps for both participants.

**Fig. 9:**
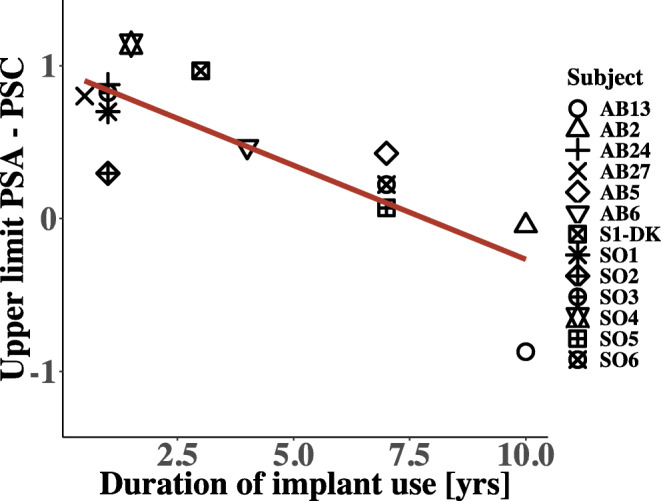
Relationship between duration of cochlear implant use in years and the log-transformed upper limit of temporal pitch (PSA-PSC). Each symbol represents one listener, where SO1–SO6 show those listeners who participated in the previous experiment (Macherey et al. 2011)

As performance changed over time for these listeners, it was investigated whether age or duration of deafness, defined here as the time between obtaining a hearing aid and receiving the implant, correlated with the effect of PSA stimulation on the upper limit. Those correlations were not significant (*p* = 0.68 and 0.98, respectively). When looking at the relationship between the duration of implantation and the difference in log-transformed upper limits of PSA and PSC across listeners, a significant negative correlation was observed (*r* = − 0.87, *p* < 0. 01165). When combining the results of both studies (labeled S1–S6 by Macherey et al. (2011) and SO1–SO6 in the following), the correlation between PSA benefit and duration of CI use remained strong and highly significant (*r* = − 0.82, *p* < 0.001) and can be seen in Fig. 9. Similarly, when removing the listeners who participated in both experiments, i.e., AB02 and AB13, as well as SO1 and SO2, the correlation still remained significant (*r* = − 0.86, *p* < 0.005). A significant correlation between the duration of CI use and the benefit of PSA stimulation on the RDRs from experiment 3 was also found (*r* = 0.93, *p* < 0.01).

This relationship may seem counterintuitive at first, as longer duration of implantation is normally associated with more training and better performance. This is true particularly in terms of speech intelligibility (Blamey et al. 2012) but also for single-electrode psychophysical measures, such as the upper limit, which has been shown to increase significantly during the first months of chronic stimulation (Carlyon et al. 2019). Based on Carlyon et al. (2019) it is also unlikely that the lack of temporal information due to the fixed-rate processing of most contemporary CI stimulation strategies is the cause for the loss of a benefit of apical stimulation over time.

However, the decrease of the benefit from PSA stimulation with longer duration of implantation is consistent with the hypothesis that insertion trauma and inflammation directly occurring after cochlear implantation may potentially facilitate neural degeneration, particularly at the apex. This hypothesis stems from observations made in cadaver studies, where in unilaterally implanted recipients with bilateral symmetric hearing loss, spiral ganglion cell counts were worse at the most apical segment in the implanted ear relative to the non-implanted ear (Khan et al. 2005; Xu et al. 2012; Seyyedi et al. 2013). Khan et al. (2005) suggested that neural degeneration in the implanted ear, which can be facilitated by insertion trauma, might be alleviated at the more basal segments through the direct electrical stimulation from the implant. Considering that PSA pulses stimulate very apical cochlear sites, the decrease in benefit of PSA stimulation over time might be a result of changes in local neural survival. Assuming that the cochlear apex receives less direct stimulation than sites located close to the stimulating electrodes, consequences similar to those associated with the effect of auditory deprivation may occur, which has been shown to lead to reduced phase-locking in the IC (Vollmer et al. 2005, 2017). Further, reduced temporal responses in the IC seen in long-term deafened animals have been shown to be most severe at IC depths that correspond to low characteristic frequencies (Middlebrooks 2018).

Another possible reason for differences in performance across individuals might arise from differences in the insertion depths of the electrode array. On one hand this could be due to the array not being inserted deeply enough to activate the apical pathway for some subjects, even with PSA stimulation. Alternatively, it might be that some subjects have a better match between the place of excitation and the pulse rate than others. Direct comparisons between apical PSA stimuli and acoustic stimuli with two CI users who had normal hearing on the contralateral ear have shown that the pitch elicited with apical PSA stimulation was either 811 or 976 Hz (Macherey et al. 2011). Thus PSA stimulation might result in similar place and rate code which has been suggested to be important for the correct representation of pitch (Oxenham et al. 2004).

A potential consequence of the apparent across-subject variability could be that experimental processing strategies incorporating more apical stimulation using pseudomonophasic pulses or phantom stimulation have not revealed any improvement for speech perception thus far (Carlyon et al., 2014). Hence, despite promising results from animal models and single-electrode psychophysics, and despite some listeners showing substantial benefit from this type of stimulation, the average effect may remain insignificant. Discovering sources of variability and finding good predictors for performance is of high importance for future research. As some patients show better performance in this study when recently implanted, perhaps those participants may have better neural survival and may eventually profit from speech processors that provide fine temporal information.

### Summary of the Findings

It has been suggested that a high-temporal-acuity brainstem pathway can be triggered when stimulation originates from apical, rather than basal, cochlear sites (Middlebrooks and Snyder 2010; Middlebrooks 2018). This study investigated the effect of stimulating more apically compared to standard methods of CI stimulation, using both the phantom-electrode mode of stimulation and pseudomonophasic anodic-first (PSA) pulse trains. Temporal processing was assessed by determining the upper limit of temporal pitch and rate discrimination ratios in three separate experiments. The following points summarize the results.

1. In agreement with previous studies (Saoji and Litvak 2010; Macherey and Carlyon 2012; Saoji et al. 2013), phantom electrode stimulation can elicit a place pitch lower than that of the most apical electrode in monopolar mode.
2. Phantom stimulation did not increase the upper limit of temporal pitch. One possible explanation is that with phantom stimulation, the side lobe on E3 is large enough to recruit enough fibers from more basal sites that may disrupt place-pitch coding.
3. PSA pulse trains could elicit a place pitch lower than that of the inverted-polarity stimulus (PSC), similar to results from previous studies (Macherey et al. 2011; Macherey and Carlyon 2012).
4. PSA pulse trains caused a substantial increase in the upper limit of temporal pitch for some subjects and slopes after 300 pps showed, on average, a significantly greater increase using this type of stimulation. However, the upper limit measured from the knee-point of the pitch-ranking function did not differ significantly between PSA and PSC stimulation. A previous study had observed a significant difference, and this remained significant when the results of the two studies were combined.
5. A small but significant effect of apical stimulation for both phantom stimulation and PSA pulse trains was found on high-rate discrimination ratios. No effect of apical stimulation was found on rate discrimination ratios when using a low-rate standard.
6. A post hoc analysis revealed a significant negative correlation between the time of implantation and the PSA benefit, which may indicate that the benefit of apical stimulation might decline over time since implantation.
